# Pre-clinical Medical Student Generated Formative Questions Can Inform the Topics Covered in Faculty Review Sessions Prior to Summative Assessments

**DOI:** 10.1007/s40670-025-02333-8

**Published:** 2025-02-26

**Authors:** Jarod Karom, Corinne Stanforth, Laura M. Banks, Jason L. Hirsch, Samuel N. Paul, Jose A. Bazan, Sheryl Pfeil, Christopher R. Pierson

**Affiliations:** 1 The Ohio State University College of Medicine, Columbus, OH 43210 USA; 2 Department of Internal Medicine, The Ohio State University College of Medicine, Columbus, OH 43210 USA; 3 Department of Biomedical Education and Anatomy, Division of Anatomy, The Ohio State University College of Medicine, Columbus, OH 43210 USA; 4 Department of Pathology, The Ohio State University College of Medicine, Columbus, OH 43210 USA; 5 Department of Pathology and Laboratory Medicine, Nationwide Children’s Hospital, Columbus, OH 43205 USA

**Keywords:** Co-creation, Formative practice, Preclinical curriculum, Review session

## Abstract

Review sessions are challenging for preclinical medical school faculty to prepare due to year-to-year variability of the strengths and weaknesses of different classes. To optimize review session planning, we piloted a novel, co-creation-based workflow within a student-faculty partnership which was well received by faculty in terms of feasibility and utility.

Review sessions prior to summative assessments are valuable endeavors, but variability year-to-year between classes can make it difficult for faculty to discern challenging topics that require more instructional time from those that require less. Resources incorporating targeted teaching based on identified knowledge deficiencies can improve learning efficiency, particularly through the use of formative multiple-choice questions (FMCQs), as practice testing is correlated with improvement in student performance [1, 2]. However, faculty time is limited in developing these learning opportunities. To meet the need for accessible, no-cost FMCQs that align with our preclinical curriculum, we built Professor-Reviewed Exam Practice (PREP), a student-led, faculty-guided partnership where students create FMCQs that are reviewed by faculty prior to distribution to the class as preparation for pre-clinical summative assessments [3]. Due to positive student feedback [3], PREP explored whether its workflow could be adapted to guide faculty in developing more targeted, efficient review sessions held before summative assessments by providing pre-session FMCQ quiz performance results to faculty to guide review session planning.

This pilot selected four review sessions based on content difficulty and previous faculty experience with PREP (Table 1). Following a meeting with review session faculty to communicate expectations, PREP drafted six to eight FMCQs per session according to PREP guidelines [3]. FMCQs were derived from curricular material presented within the preceding 4–6 weeks. Each FMCQ was drafted in a USMLE vignette-style and peer-reviewed before faculty review. Faculty evaluated relevance and accuracy of each FMCQ, with recommended edits implemented prior to release to the class. FMCQs were released to the class as a Google Forms Quiz (Alphabet Inc, Googleplex, Mountain View, CA), which permitted anonymous monitoring of student participation and collective performance data. Faculty could choose how to utilize results either by incorporating FMCQs into their review session for discussion or by using quiz performance results to guide allocation of time to review specific topics. FMCQs were released to the class 7 days before the review session and available until the day before the review session, with performance results shared with faculty thereafter.

**Table 1:** Curricular review sessions, number of formative multiple-choice questions (FMCQs) per quiz, average quiz score, and student participation rates

Curricular review sessions ^†^	Number of FMCQs	Average quiz score (percentage)	Number of participating students (percent of class)
Foundational Histology (Foundations 2 Block)	6	71	69 (35%)
Clinical Gastroenterology (Gastrointestinal & Renal Disorders Block)	8	64	33 (17%)
Gastrointestinal Histology (Gastrointestinal & Renal Disorders Block)	8	54	27 (14%)
Clinical Infectious Disease (Host Defense Block)	7	65	34 (17%)

^†^Review sessions appear chronologically as they occurred in the curriculum

Participating faculty (*n* = 4) were surveyed regarding use of this pilot in tailoring their review session. All faculty reported using the PREP FMCQ results to guide review session planning, and all were interested in utilizing the workflow in the future. When asked, “How PREP FMCQ results contributed to the structure of your review session?” the following comments were shared:

The PREP questions allowed me to better integrate histology and pathology together along topics that were identified as challenging for students. I did not have to guess which topics were challenging.

It allows faculty to better appreciate the difficult concepts from class-to-class and strategically re-teach those concepts.

The results of the quiz helped inform the review session. I think this is a valuable endeavor—especially sending the results to the block leader … the results were summarized in a readable and understandable manner.

This pilot tested the feasibility and faculty acceptance of a workflow that generates FMCQs to help guide the development of curricular review sessions prior to high-stakes summative assessments. We considered faculty buy-in as critical to success, so exploring their perspectives as a first test of this workflow was prioritized. All faculty partners expressed enthusiasm and willingness to continue using this workflow in the future. Feedback indicates that this workflow’s strength lies in permitting faculty a better sense of time allocation for content review, allowing emphasis on key topics identified as more difficult among the class. It is possible that this workflow could also inform the design of other curricular elements, including small group and problem-based learning sessions.

Limitations to this pilot include the (1) small number of review sessions in which the workflow was implemented; (2) relatively low student participation in PREP quiz completion (Table 1); and (3) anonymous student participation, which prevented us from correlating PREP quiz completion and review session attendance and collecting student feedback, although positive feedback was previously collected [3]. Nevertheless, our survey demonstrated that all participating faculty found the FMCQ data helpful in planning review sessions, and we believe that with continued student-faculty collaboration and incorporation of this workflow, student participation rates will increase.

This study introduces a novel co-creation-based workflow where students and faculty collaborate to tailor review sessions with the shared goal of improved efficiency and enhanced learning opportunities. Future directions will focus on continued refinement based on faculty preferences and the extension of this workflow to guide planning of other curricular components.
